# Para-duodenal hernia: a report of five cases and review of literature

**DOI:** 10.1186/s12893-018-0365-8

**Published:** 2018-05-30

**Authors:** Kamleshsingh Shadhu, Dadhija Ramlagun, Xiaochun Ping

**Affiliations:** 1 Department of General Surgery, Jiangsu Province Hospital, First Affiliated hospital of Nanjing Medical University, Guangzhou Road, 300, Gulou District, Nanjing, 210029 Jiangsu Province People’s Republic of China; 2 Pancreas Center, Jiangsu Province Hospital, First Affiliated hospital of Nanjing Medical University, Guangzhou Road, 300, Gulou District, Nanjing, 210029 Jiangsu Province People’s Republic of China; 3 Department of Breast Surgery, Jiangsu Province Hospital, First Affiliated hospital of Nanjing Medical University, Guangzhou Road, 300, Gulou District, Nanjing, 210029 Jiangsu Province People’s Republic of China; 4 Department of Gastric Surgery, Jiangsu Province Hospital, First Affiliated hospital of Nanjing Medical University, Guangzhou Road, 300, Gulou District, Nanjing, 210029 Jiangsu Province People’s Republic of China

**Keywords:** Paraduodenal hernia, Intestinal obstruction, Abdominal pain, CT images, Laparoscopic surgery

## Abstract

**Background:**

Para-duodenal hernia (PDH) represents rare clinical entities based on few literatures.

**Case presentation:**

We report five cases of Para-duodenal hernia, all occurring in male patients ranging from 34 to 75 years of age. The patients had varied manifestations presenting with abdominal pain with or without vomiting and nausea and with or without signs of intestinal obstruction. CT images showed cluster of dilated bowel segments with displaced mesenteric vessels at hernial orifice. Laparoscopic surgical approach was adopted, and the patients were discharged about a week later without further complications.

**Conclusion:**

We hope to raise awareness about the management of this rare clinical entity and the benefits of CT imaging and laparoscopic surgery as standard approaches.

## Background

Para-duodenal hernias (PDH) have traditionally been considered the most frequent type of congenital internal hernia [1]. Left para-duodenal hernia (hernia of Lanzert) is about three times more common than the right counterpart (Waldayer’s hernia) [2]. Left para-duodenal hernia (LPDH) is a congenital defect with an occurrence of approximately 2% of the population. It is posterior to the inferior mesenteric vein and left branches of middle colic artery and is situated to the left of the fourth part of the duodenum. It arises from the fossa of Landzert [3–5]. The fossa to the left of the fourth part of the duodenum is the area where the small bowel loops (usually jejunum) prolapse through and into the left portion of the transverse mesocolon. The herniated small bowel loops may therefore become trapped within the mesenteric sac [5, 6]. The initial rotation of the midgut behind and then left to the superior mesenteric artery and comes to lie in the left side of the abdomen behind the mesentery of the descending colon leads to development of LPDH [7]. The right para-duodenal hernia (RPDH) occurs when the small bowel herniates through a defect in the first part of the jejunal mesentery in the so called Waldeyer’s fossa. At autopsy, the Waldeyer’s fossa was found in about 1% of the population [8]. The malrotation of the midgut and failure of fusion of mesentery to parietal peritoneum create a hernial defect called RPDH. PDH can lead to bowel obstruction, ischemia, and perforation with a high mortality [6]. Clinical diagnosis of PDH is a challenge as symptoms are entirely non-specific. They usually affect males more than females (3:1) [9, 10]. Most patients are diagnosed between the 4th and 6th decades of life and the mean age of diagnosis is 38.5 years [2]; for our case series all were males. 75% of mesocolic hernias occur on the left side and 25% on the right side with middle mesocolic hernia being very rare [11]. In medical literature, para-duodenal hernias causing intestinal obstruction are few and report no evidence of long lasting postoperative ileus after surgery. We report herein five cases of PDH and their management based on a review of literatures.

## Case presentation

### Case 1

**Fig. 1:**
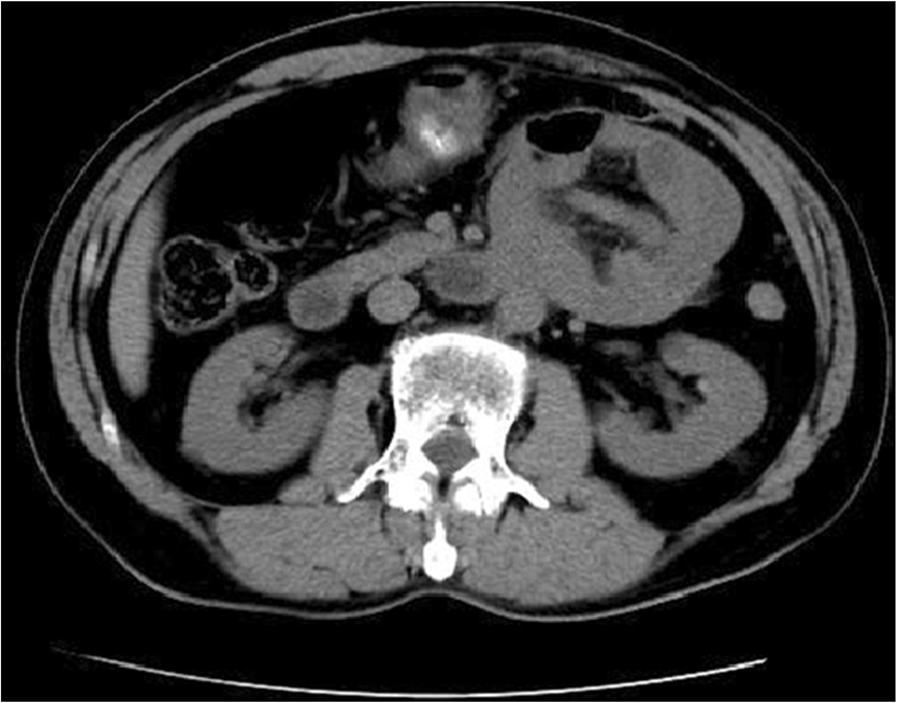
Transverse view of CT image showing the cluster of small intestine and its mesentery. The entrapped intestinal loop was behind the infernal mesenteric vein (white arrow)

**Fig. 2:**
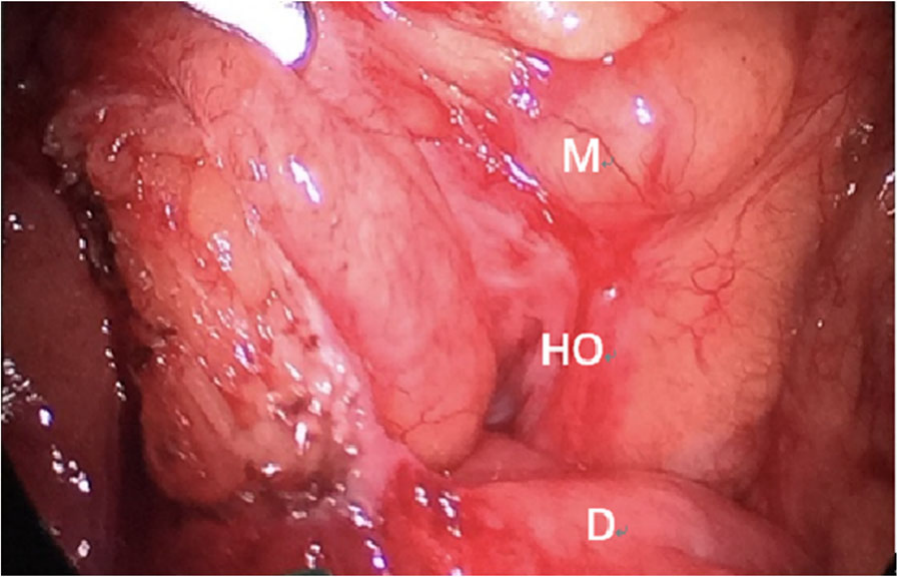
The defect in the transverse mesocolon. M: transverse mesocolon, D: the ascending part of duodenum, HO: hernia orifice

A 68-year-old man complained left middle abdominal pain for 10 days. The pain was paroxysmal without nausea and vomiting. It got worse after meals. He has a past medical history of chronic gastritis treated with regular proton pump inhibitor. He had laparoscopic cholecystectomy 2 years ago. The results of complete blood count, serum and urine amylase were within normal limit. Endoscopy showed chronic gastritis with bile reflux. His abdominal CT scan showed that a part of small intestine and its mesentery were folded together on the left side of the abdomen (Fig. 1). The patient was diagnosed with abdominal internal hernia and laparoscopic surgery was done. During the surgery, a soft mass of 5 cm in diameter was found within the transverse mesocolon. It was later found to be that 60 cm of the small intestine, which was trapped through a defect in the transverse mesocolon. The defect was 2 cm in diameter on the left side of the ascending part of duodenum (Fig. 2) within the mesocolon. The entrapped intestinal loop was reduced, and the defect was repaired. He was diagnosed with left paraduodenal hernia and was discharged on 7th post-operative day. No abnormal presentation was found during follow-up. (Table 1).

**Table 1:** Summary of five cases of paraduodenal hernia

Cases	1	2	3	4	5
Features					
Age (years old)	68	34	68	75	40
Gender	Male	Male	Male	Male	Male
Imaging modality	CT	CT	CT	CT	X-ray, CT
Radiological signs	CT scan showed a part of small intestine and its mesentery folded together at the left abdomen.	CT scan showed a fold at the left upper abdomen, consisting of small intestine coiled with mesentery.	CT scan showed part of small intestine was folded.	CT scan showed jejunum was folded at the left abdomen.	X-ray showed a mass at right upper abdominal quadrant. CT scan showed a part of small intestine and its mesentery were folded together at the right abdomen.
Location of hernia	Left	Left	Left	Left	Right
Presenting symptoms	Left middle abdominal paroxysmal pain for 10 days without nausea and vomiting.	Left upper abdominal paroxysmal pain for 24 h with vomiting.	Upper abdominal paroxysmal pain for 2 days with vomiting.	Paraumbilical pain and abdominal distention with nausea and vomiting for 13 h.	Right abdominal pain for 11 h with sudden onset.
Type of surgery	Laparoscopy	Laparoscopy	Laparoscopy	Laparoscopy turned into open	Laparoscopy
Discharge (n^th^ POD)	7	7	7	14	9
Complications during follow-up	None	None	None	None	None

### Case 2

**Fig. 3:**
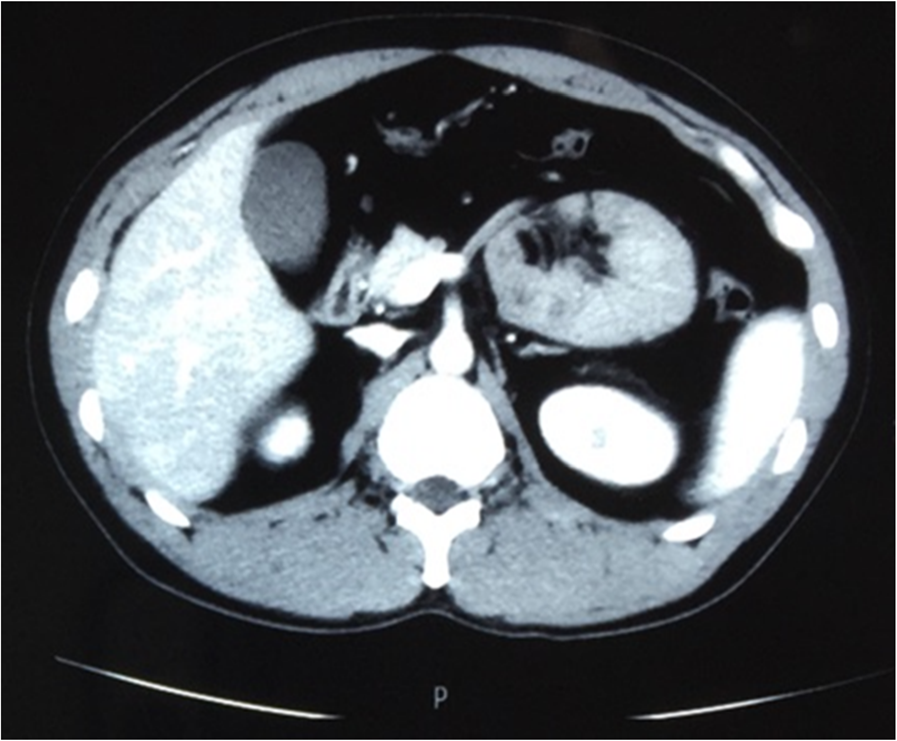
Transverse view of CT image showing the cluster of small intestine and its mesentery

**Fig. 4:**
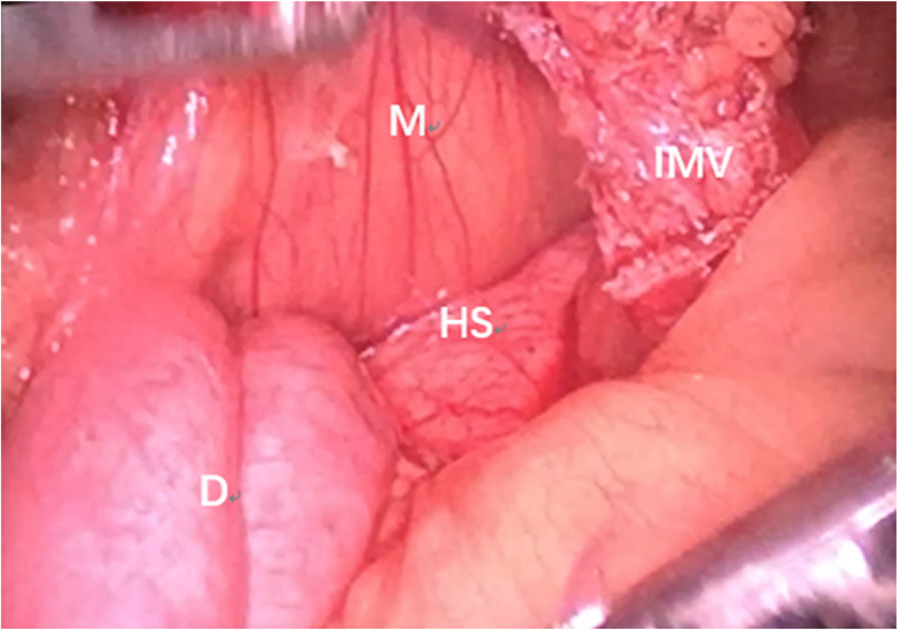
The defect in the transverse mesocolon was opened. M: mesocolon, IMV: inferior mesenteric vein, D: the ascending part of duodenum, HS: hernia sac

A 34-year-old man complained about left upper abdominal pain for 24 h. The pain was paroxysmal accompanied with vomiting. His abdominal CT scan showed a fold, at the left upper abdomen, consisting of small intestine coiled with mesentery (Fig. 3). Subsequent laparoscopy found a loop of jejunum was entrapped in the left side of mesocolon through a defect on the left side of the ascending part of duodenum. After the reduction of the small intestine, the hernia orifice was opened large enough to prevent further herniation (Fig. 4). Post-operative recovery was uneventful, and the patient was discharged on 7th post-operative day. No abnormalities were found during follow-up so far. (Table 1).

### Case 3

**Fig. 5:**
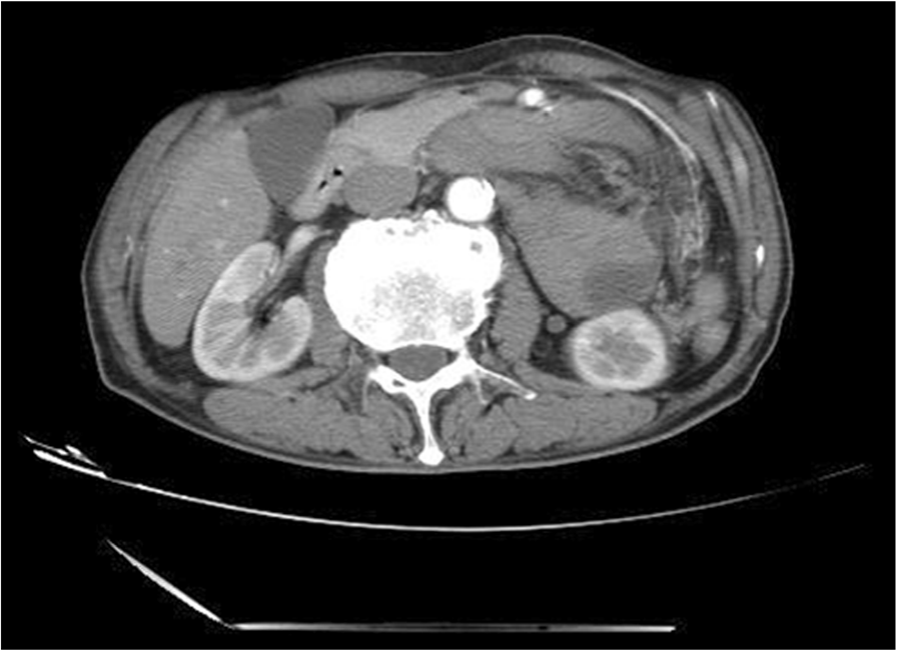
Transverse view of CT image showing the cluster of small intestine and its mesentery entrapped in the left side of abdomen, beneath the left branch of middle mesenteric artery. The attenuated enhancement of the entrapped intestinal loop suggested intestinal ischemia

**Fig. 6:**
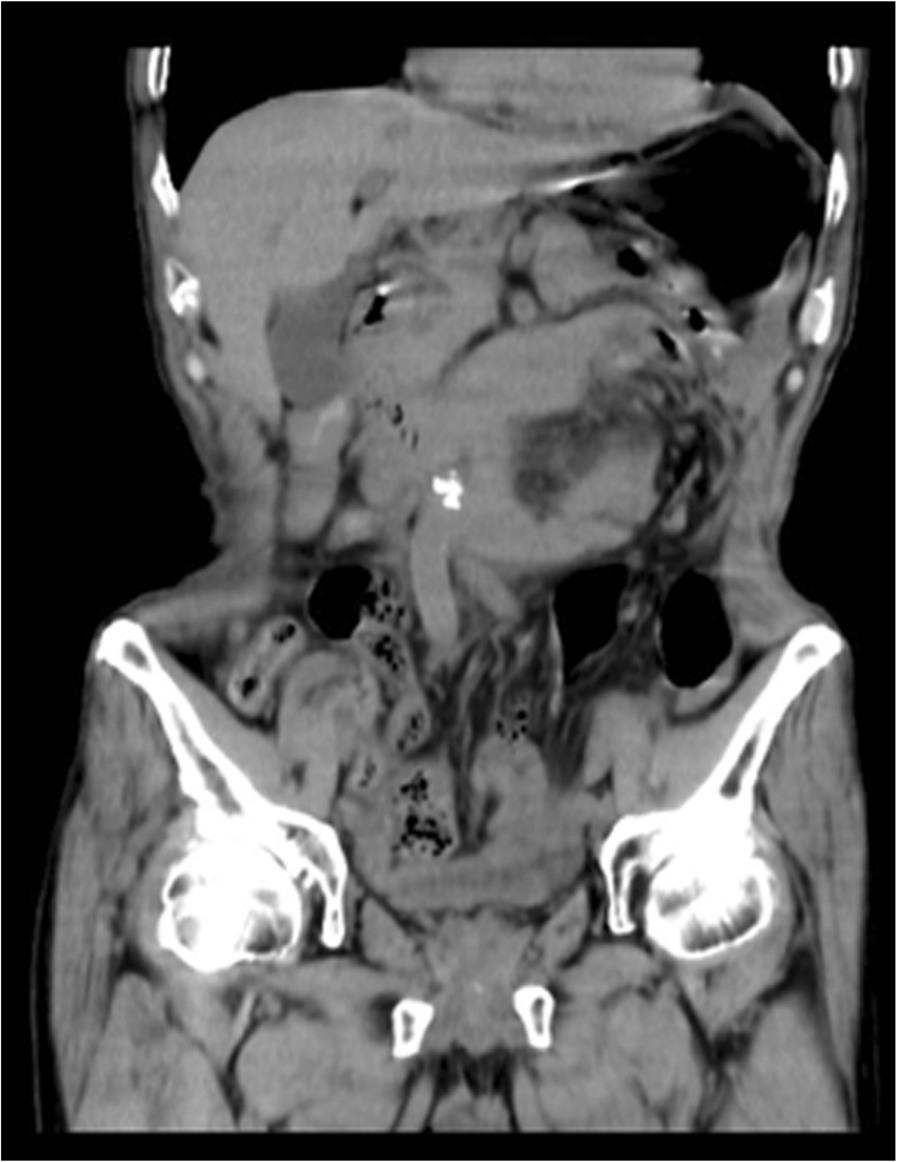
Coronary view of CT scan image showing the cluster of the small intestine

**Fig. 7:**
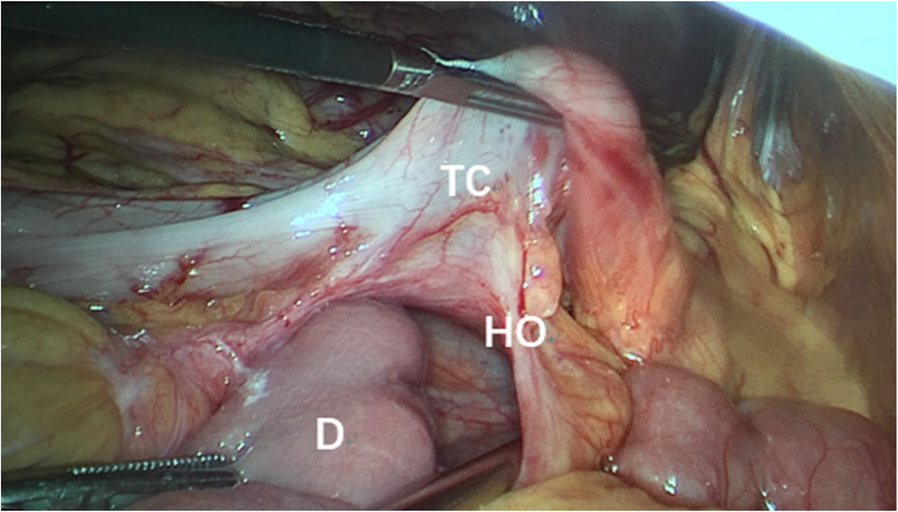
The defect in the transverse mesocolon. TC: transverse colon, D: the ascending part of duodenum, HO: hernia orifice

A 68-year-old man complained about upper abdominal pain for 2 days. The pain was paroxysmal accompanied with vomiting. He was diagnosed with chronic gastritis 2 months ago and was treated with medications without any relief. His abdominal CT imaging showed part of the small intestine was folded (Figs. 5, 6). He underwent paraduodenal hernioplasty via laparoscopy. Surgery consisted of reduction of intestinal loops in the hernial sac and subsequent repair of the defect (Fig. 7). The length of the folded intestine was found to be 100 cm and the hernia was on the left side of mesocolon. He was discharged on 7th post-operative day and had no complications during follow-up so far. (Table 1).

### Case 4

**Fig. 8:**
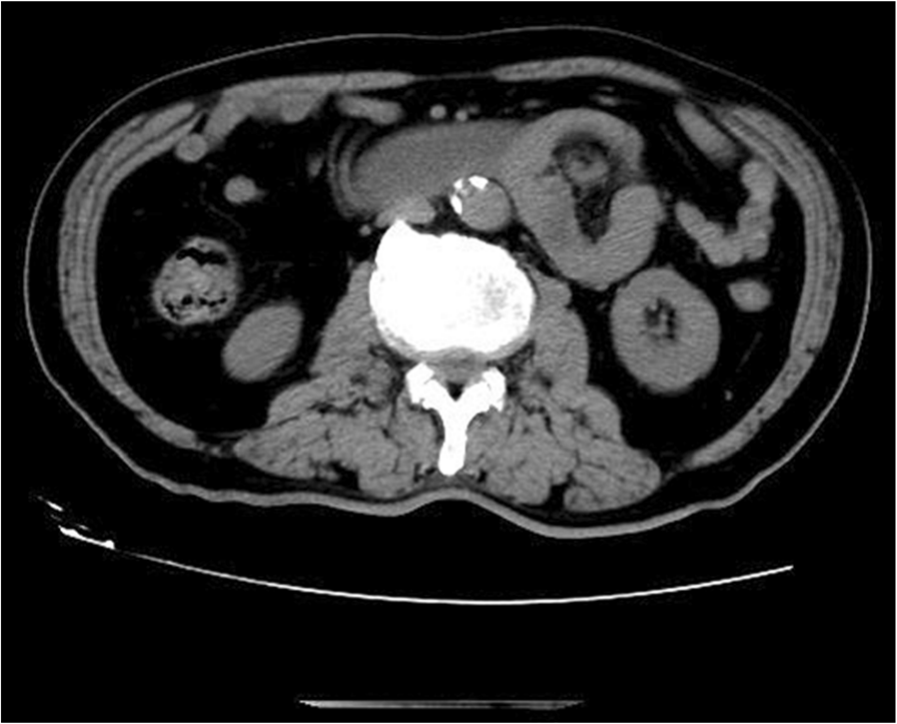
Transverse section of CT scan image showing the cluster of jejunum

**Fig. 9:**
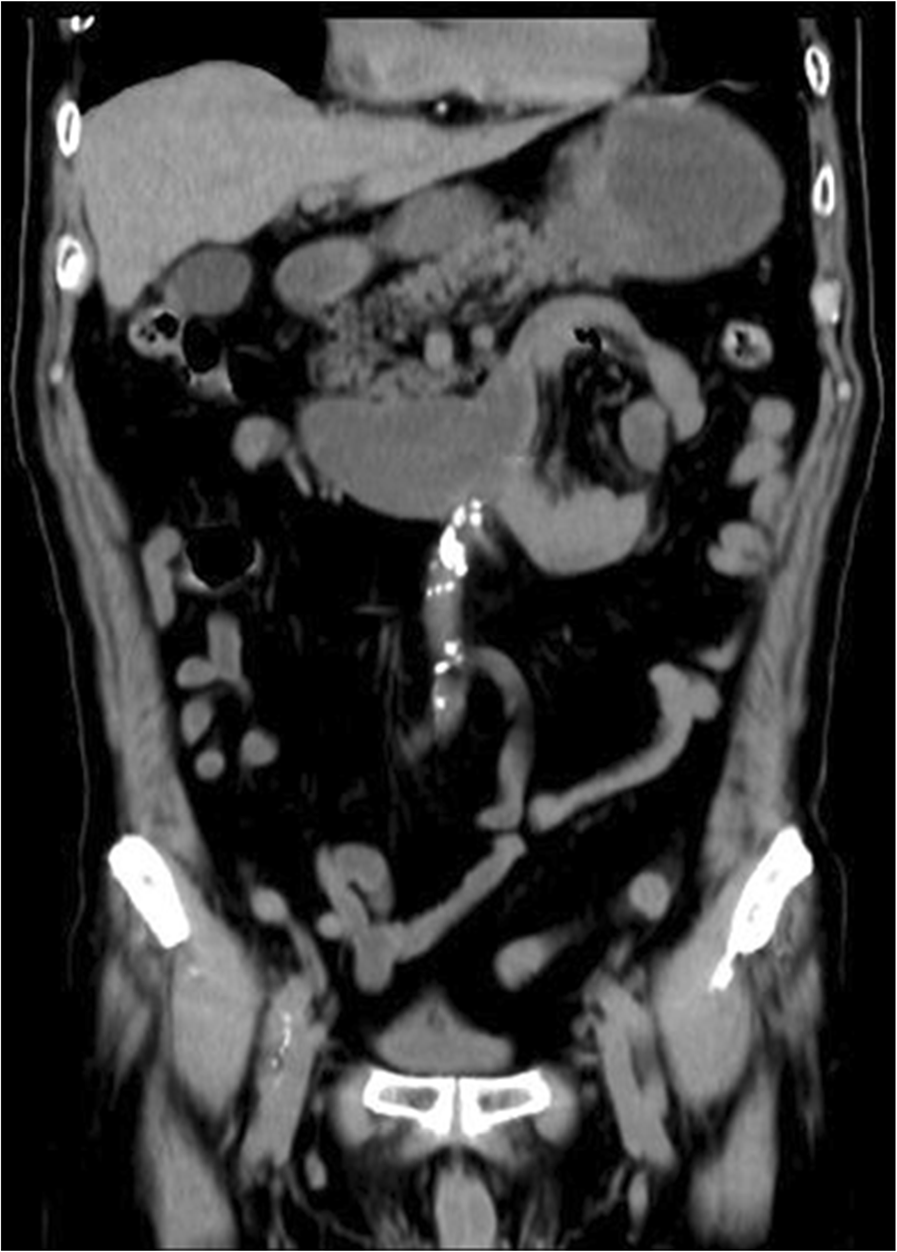
Coronary view of CT scan image showing the cluster of the small intestine

**Fig. 10:**
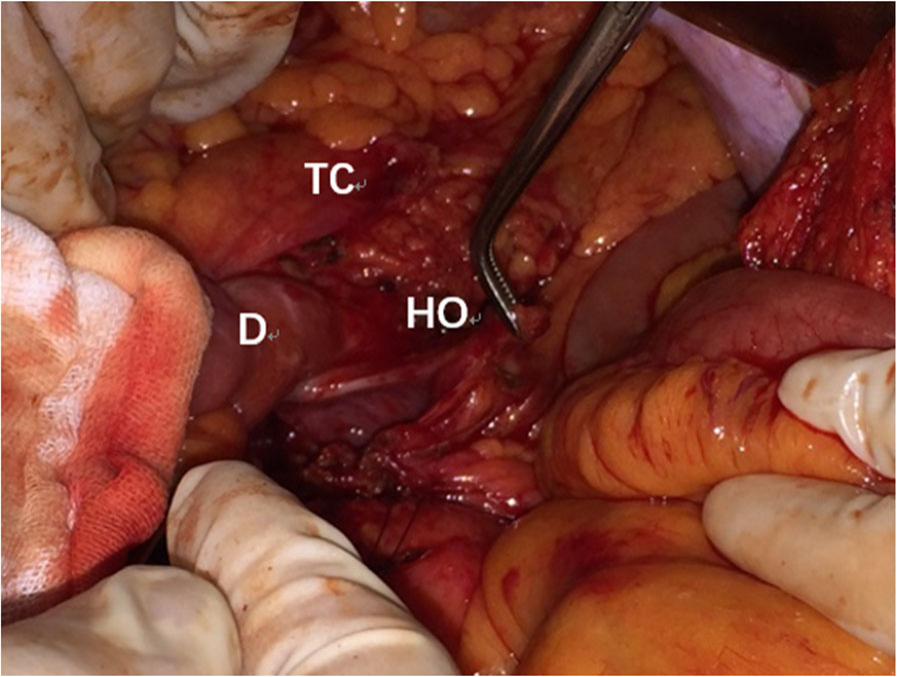
The defect of transverse mesocolon was identified and closed with interrupted sutures. TC: transverse colon, D: the ascending part of duodenum, HO: hernia orifice

A 75-year-old man complained about abdominal distention and pain for 13 h. The pain was paraumbilical accompanied with nausea and vomiting. His abdominal CT imaging showed that the jejunum was folded at the left abdomen (Figs. 8, 9). He was diagnosed with left paraduodenal hernia and laparoscopy was carried out. During the surgery, reduction of the entrapped jejunal loops from the hernial sac was attempted but failed due to severe adhesion with surrounding organs. It was then converted to open enterolysis. The defect of the mesocolon was found on the right side of the ascending duodenum and was repaired eventually with interrupted sutures (Fig. 10). After the surgery, the patient still felt occasional distension after meals, but no acute episode of obstruction has ever occurred. He was discharged two weeks later, and he had no complications during follow-up so far. (Table 1).

### Case 5

**Fig. 11:**
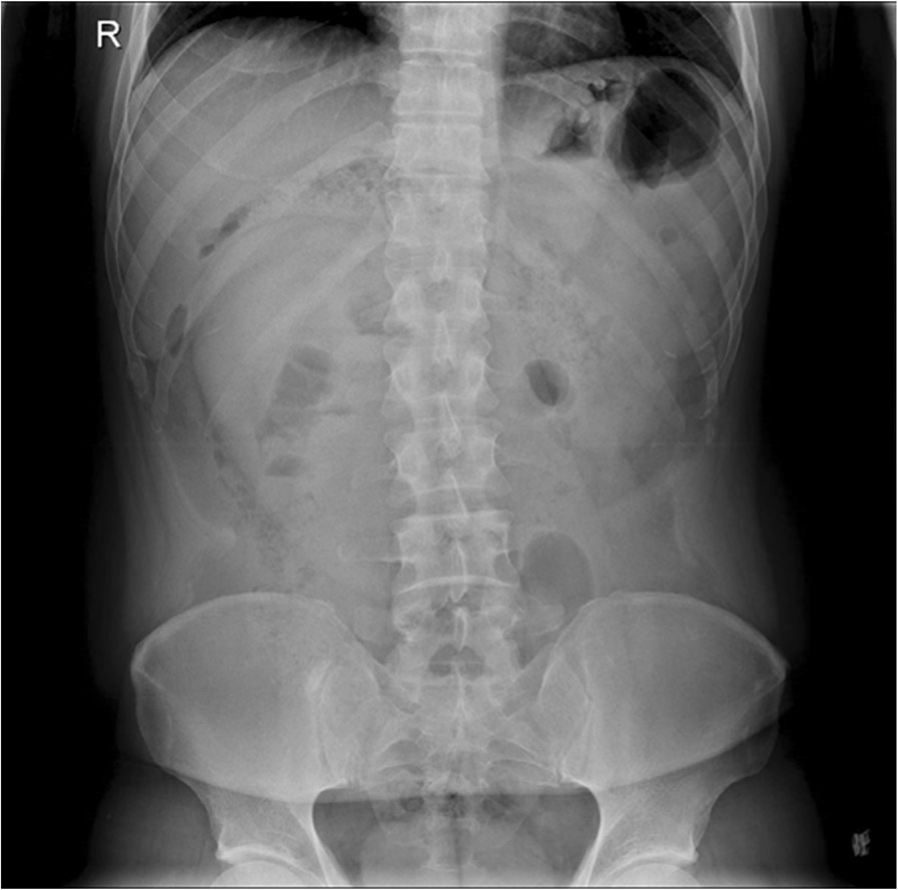
Abdominal X-ray showed a mass in the right upper quadrant of abdomen

**Fig. 12:**
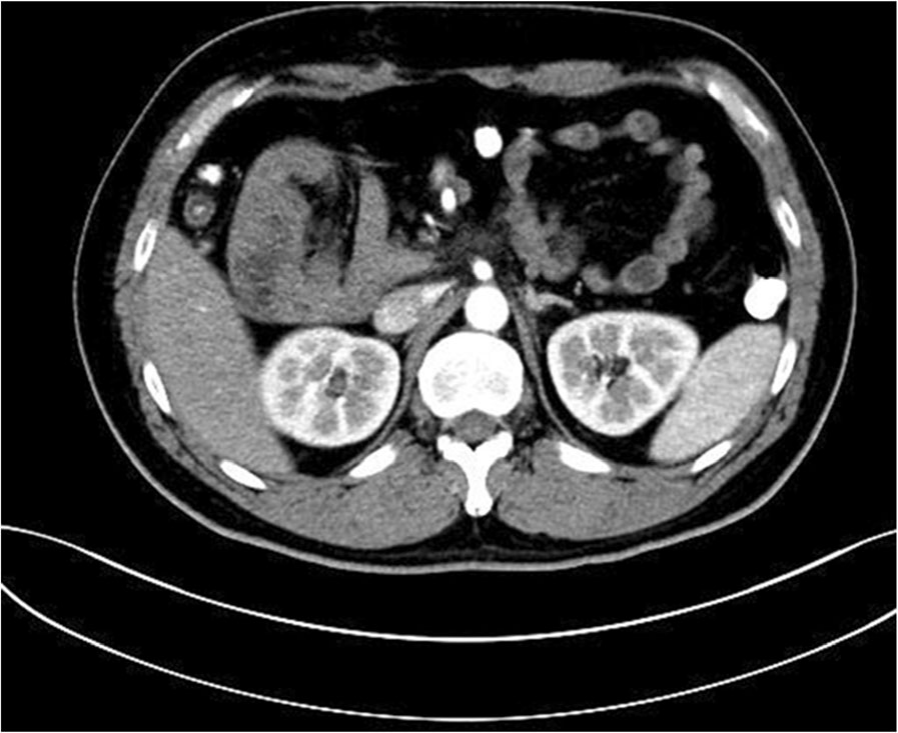
Transverse view of CT image showing the cluster of small intestine and its mesentery on the right upper quadrant of abdomen before the descending part of duodenum

**Fig. 13:**
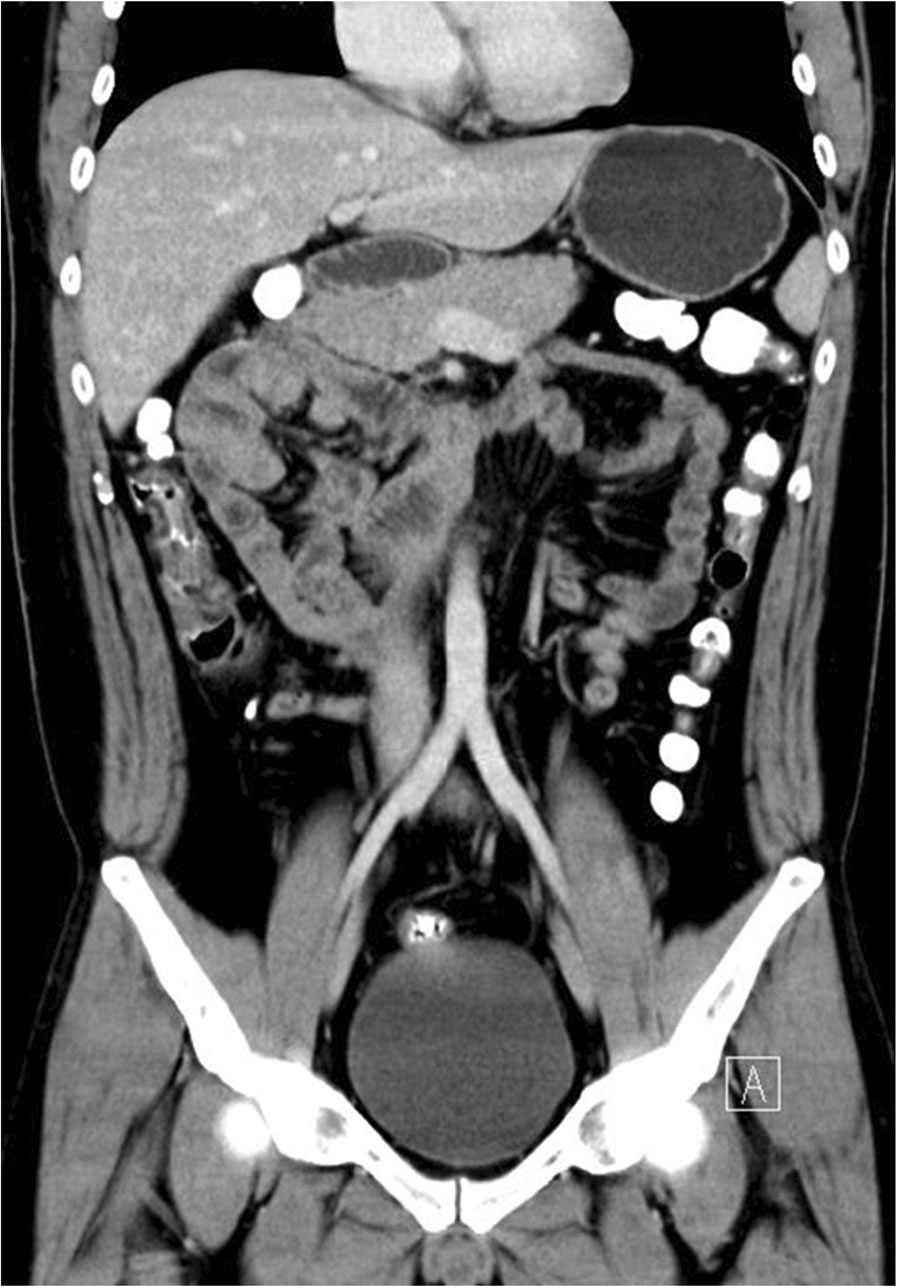
Coronary view of CT image showing the cluster of small intestine and its mesentery trapped within the right side of mesocolon

A 40-year-old man complained about right abdominal pain for 11 h. The pain occurred suddenly without triggering factors. It was mild and intermittent. He has a history of laparoscopic cholecystectomy for one year. His abdominal X-ray showed a mass in the right upper quadrant of abdomen (Fig. 11). CT scan showed that a part of small intestine and its mesentery were folded together at the right abdomen (Figs. 12, 13). A diagnosis of right paraduodenal hernia was made. The patient underwent laparoscopic surgery during which the entrapped intestinal loop was reduced from a defect in the first part of the jejunal mesentery. He was discharged on 9th post-operative day. No other abnormal presentation was found during follow-up so far. (Table 1).

## Discussion & conclusion

PDH also known as mesocolic hernias are congenital and derive from embryonary peritoneal anomalies and associated abnormal intestinal rotation [12]. These patients usually present with chronic abdominal pain and vomiting with or without signs of intestinal obstruction [9]. There is an associated risk of strangulation and intestinal infarction for more than 50% over the course of a lifetime, making it necessary to investigate radiological signs of hypoperfusion and intestinal ischemia [13]. The high rate of mortality associated with these complications make early identification indispensable and justifies the role of abdominal CT in the early pre-operative diagnosis of paraduodenal hernia. Multislice computer tomography (CT) offers high resolution and multiplanar images which may be very demonstrative and characteristic providing a precise and early diagnosis, useful for surgical treatment planning [6, 12]. In typical CT images, PDH shows a cluster of dilated bowel segments with engorged and displaced mesenteric vessels at the hernial orifice [14]. Early surgical intervention is essential to avoid future complications because patients with PDH have a 20–50% mortality for acute presentations [15, 16]. A literature search was performed to identify the rare cases of paraduodenal hernia treated with laparoscopic approach. Only 28 case reports were published between January 1998, in which Uernatsu et al. [17]. first described the minimally invasive treatment of this surgical emergency, and November 2015. The several advantages of laparoscopic approach, deduced after analysing data in previous cases, were: decrease in post-operative pain, reduced morbidity, early food resumption (1.33 average, 1–3), shorter hospital stay (3.60 average, range 1–10). These benefits occurred regardless of type of intervention (elective or emergency), type of repair (closure of hernial defect with continuous or interrupted suture, enlargement of defect or resection of the sac) and type of material used (adsorbable or not adsorbable, monofilament or poly-filament) [9, 10, 17–35]. We opted for laparoscopic approach for all our five patients who shared same benefits as described by other authors.

Therefore, based on our experience and current literatures, we believe that laparoscopic approach is the optimum treatment strategy for patients with paraduodenal hernia, especially in health centres with strong experience of advanced laparoscopic surgery. Patients with or without small bowel obstruction and hemodynamically stable are suitable to enjoy the benefits of laparoscopic approach. Computed tomography remains the gold standard of imaging modality for early diagnosis of this clinical entity. However, the limitation of our report is that the number of cases is small and hence, a concrete conclusion can only be drawn based on our experience so far.

## Abbreviations

CT: Computed Tomography
LPDH: Left paraduodenal hernia
PDH: Paraduodenal hernia
POD: Post-operative Day
RPDH: Right paraduodenal hernia

## References

[1] Blachar A, Federle MP, Dodson SF (2001). Internal hernia: clinical and imaging findings in 17 patients with emphasis on CT criteria. Radiology.

[2] Khan MA, Lo AY, Vande Maele DM (1998). Paraduodenal hernia. Am Surg.

[3] Husain A (2012). Internal Hernia through Paraduodenal Recess with Acute Intestinal Obstruction: A Case Report. Indian J Surg.

[4] Zonca P (2008). Treitz’s hernia. Hernia.

[5] Armstrong O (2007). Internal hernias: anatomical basis and clinical relevance. Surg Radiol Anat.

[6] Martin LC, Merkle EM, Thompson WM (2006). Review of internal hernias: radiographic and clinical findings. AJR Am J Roentgenol.

[7] Bartlett MK, Wang C, Williams WH (1968). The surgical management of paraduodenal hernia. Ann Surg.

[8] Hassani KI (2014). Left paraduodenal hernia: A rare cause of acute abdomen. Pan Afr Med J.

[9] Assenza M (2014). Laparoscopic management of left paraduodenal hernia. Case report and review of literature. G Chir.

[10] Palanivelu C (2008). Laparoscopic management of paraduodenal hernias: mesh and mesh-less repairs. A report of four cases. Hernia.

[11] Kannan NS (2014). Congenital middle mesocolic hernia: A case report. Australas Med J.

[12] Takeyama N (2005). CT of internal hernias. Radiographics.

[13] Ross D, Cawich SO (2010). A case of a paraduodenal hernia. Int J Surg Case Rep.

[14] Blachar A (2001). Radiologist performance in the diagnosis of internal hernia by using specific CT findings with emphasis on transmesenteric hernia. Radiology.

[15] Al-Khyatt W (2013). Acute intestinal obstruction secondary to left paraduodenal hernia: a case report and literature review. World J Emerg Surg Wjes.

[16] Socas MM (2006). Atypical left paraduodenal hernia. Rev Esp Enferm Dig.

[17] Uematsu T (1998). Laparoscopic repair of a paraduodenal hernia. Surg Endosc.

[18] Moon CH, et al. Diagnostic Laparoscopy and Laparoscopic Repair of a Left Paraduodenal Hernia Can Shorten Hospital Stay. J Soc Laparoendosc Surg. 2006;10(1):90–3. PMC3015685 16709368

[19] Uchiyama S (2009). An unusual variant of a left paraduodenal hernia diagnosed and treated by laparoscopic surgery: report of a case. Surg Today.

[20] Finck CM (2000). A novel diagnosis of left paraduodenal hernia through laparoscopy. Surg Endosc.

[21] Rollins MD, Glasgow RE (2004). Left paraduodenal hernia. J Am Coll Surg.

[22] Fukunaga M (2004). Laparoscopic surgery for left paraduodenal hernia. J Laparoendosc Adv Surg Tech A.

[23] Tuyoshi S (2007). Left Paraduodenal Hernia Successfully Treated with Laparoscopic Surgery: A Case Report. Case Rep Gastroenterol.

[24] Jeong GA (2008). Laparoscopic repair of paraduodenal hernia: comparison with conventional open repair. Surg Laparosc Endosc Percutan Tech.

[25] Poultsides GA, et al. Image of the Month–Quiz Case. Arch Surg. 2009;144(3):287–88. doi:10.1001/archsurg.2008.551-a

[26] Khalaileh A (2010). Left laparoscopic paraduodenal hernia repair. Surg Endosc.

[27] Parmar BPS, Parmar RS (2010). Laparoscopic management of left paraduodenal hernia. J Minim Access Surg.

[28] Almufarrej F (2011). Image of the month. Left-sided paraduodenal hernia. Arch Surg.

[29] Nam SH (2012). Laparoscopic treatment of left paraduodenal hernia in two cases of children. Int J Surg Case Rep.

[30] Hussein M (2012). Laparoscopic repair of a left paraduodenal hernia presenting with acute bowel obstruction: report of a case. Surg Laparosc Endosc Percutan Tech.

[31] Siddika A, Coleman AHL, Pearson TE (2013). Laparoscopic repair of left paraduodenal hernia. J Surg Case Rep.

[32] Lee SE, Choi YS (2014). Left paraduodenal hernia combined with acute cholecystitis. Ann Surg Treat Res.

[33] Lim CH, Ong HS, Eng A (2015). A Rare Cause of Abdominal Pain. Left Paraduodenal Hernia. Gastroenterology.

[34] Winder JS, Pauli EM, Haluck RS (2015). Laparoscopic repair of a left-sided paraduodenal hernia. Surg Endosc.

[35] Milani D (2013). A case of a paraduodenal hernia with a concomitant mesosigmoid defect. Cent Eur J Med.

